# Undiagnosed cardiovascular disease prior to cardiovascular death in individuals with severe mental illness

**DOI:** 10.1111/acps.13017

**Published:** 2019-03-29

**Authors:** I. H. Heiberg, B. K. Jacobsen, L. Balteskard, J. G. Bramness, Ø. Næss, E. Ystrom, T. Reichborn‐Kjennerud, C. M. Hultman, R. Nesvåg, A. Høye

**Affiliations:** ^1^ Center for Clinical Documentation and Evaluation (SKDE) Tromsø Norway; ^2^ Department of Community Medicine UiT – The Arctic University of Norway Tromsø Norway; ^3^ Centre for Sami Health Research Department of Community Medicine UiT – The Arctic University of Norway Tromsø Norway; ^4^ Norwegian National Advisory Unit on Concurrent Substance Abuse and Mental Health Disorders Innlandet Hospital Trust Hamar Norway; ^5^ Department of Clinical Medicine UiT – The Arctic University of Norway Tromsø Norway; ^6^ Institute of Clinical Medicine University of Oslo Oslo Norway; ^7^ Institute of Health and Society University of Oslo Oslo Norway; ^8^ Department of Mental Disorders Norwegian Institute of Public Health Oslo Norway; ^9^ Department of Psychology University of Oslo Oslo Norway; ^10^ PharmacoEpidemiology and Drug Safety Research Group School of Pharmacy University of Oslo Oslo Norway; ^11^ Department of Medical Epidemiology and Biostatistics Karolinska Institutet Stockholm Sweden; ^12^ Icahn School of Medicine Mt Sinai Hospital New York NY USA; ^13^ Norwegian Medical Association Oslo Norway; ^14^ Division of Mental Health and Substance Abuse University Hospitalof North Norway Tromsø Norway

**Keywords:** schizophrenia, bipolar disorder, cardiovascular diseases, death, adult, treatment delay, delayed diagnosis

## Abstract

**Objective:**

To examine whether individuals with schizophrenia (SCZ) or bipolar disorder (BD) had equal likelihood of not being diagnosed with cardiovascular disease (CVD) prior to cardiovascular death, compared to individuals without SCZ or BD.

**Methods:**

Multivariate logistic regression analysis including nationwide data of 72 451 cardiovascular deaths in the years 2011–2016. Of these, 814 had a SCZ diagnosis and 673 a BD diagnosis in primary or specialist health care.

**Results:**

Individuals with SCZ were 66% more likely (OR: 1.66; 95% CI: 1.39–1.98), women with BD were 38% more likely (adjusted OR: 1.38; 95% CI: 1.04–1.82), and men with BD were equally likely (OR: 0.88, 95% CI: 0.63–1.24) not to be diagnosed with CVD prior to cardiovascular death, compared to individuals without SMI. Almost all (98%) individuals with SMI and undiagnosed CVD had visited primary or specialized somatic health care prior to death, compared to 88% among the other individuals who died of CVD.

**Conclusion:**

Individuals with SCZ and women with BD are more likely to die due to undiagnosed CVD, despite increased risk of CVD and many contacts with primary and specialized somatic care. Strengthened efforts to prevent, recognize, and treat CVD in individuals with SMI from young age are needed.

**Significant outcomes**

- In this nationwide study, we found a considerable under diagnosis of cardiovascular disease (CVD) prior to cardiovascular death in individuals with schizophrenia (SCZ). Similar relationships were found for women, but not men, with bipolar disorder (BD).
- The higher likelihood of undiagnosed CVD applied to all main types of cardiovascular death and was particularly pronounced in the youngest.
- Almost all individuals with SMI and undiagnosed CVD prior to cardiovascular death were in contact with primary or specialized somatic health care in the observation period before death, demonstrating that opportunities for identification and management of CVD risk factors exist.

**Limitations**

- Information on antipsychotic medications and history of CVD prior to the study period was lacking.
- As for other studies based on health registries, the diagnostic quality has not been established for all disease categories.

## Introduction

Individuals with schizophrenia (SCZ) or bipolar disorder [BD; henceforth referred to as severe mental illness (SMI)] have impaired cardiovascular health 1, 2. A higher incidence of cardiovascular disease (CVD) in individuals with SMI has been documented 3, 4, as well as increased mortality from CVD compared to the general population 5, 6. Cardiovascular mortality has been reduced by 50% in the general population since 2000 7, but remain high among individuals with SMI 8, 9. A recent study found that Norwegians with SCZ treated in specialized health care in the period 2009–2015 had a fourfold to fivefold increased risk of cardiovascular death compared to the general population 10, which is substantially higher than the doubled risk reported in earlier studies 11, 12.

Excess cardiovascular mortality in individuals with SMI is often attributed to socioeconomic disadvantage 13, lifestyle factors such as unhealthy diet, physical inactivity, smoking, and alcohol abuse 14, and metabolic side‐effects of antipsychotic medication 15. It is also a substantial overlap between genetic risk for SMI and genetic risk for type I diabetes, hypertension, cardiac dysrhythmia, and non‐rheumatic heart disease 16, indicating additional frailty for CVD in individuals with SMI. There is, however, a rising concern that suboptimal health care, including underdiagnosis and treatment delay of CVD, may also contribute to the elevated cardiovascular mortality among individuals with SMI 17, 18. Meta‐analytic studies have reported inferior preventive care regarding CVD risk factors in patients with SMI, compared to others 19, 20, 21. Large population‐based cohort studies have reported lower or similar rates of recorded CVDs such as atrial fibrillation, hypertension, and ischemic heart disease (IHD) in individuals with SMI, compared to others 1, 22, 23, 24, in spite of increased risk of CVD. Lower prescriptions rates for cardiovascular medication in individuals with SMI compared to others have also been reported 25, 26, 27, possibly implying underdiagnosis and under‐treatment of CVD in these individuals. Furthermore, in a survey of mental health service users, 39% reported not having discussed CVD risk factors with healthcare professionals the previous year 28.

While evidence regarding the prevalence of undiagnosed CVD in individuals with SMI is accumulating, mortality from undiagnosed CVD in individuals with SMI is less studied. Exceptions include two nationwide Swedish studies, where the proportion of individuals who died from IHD undiagnosed 1 month prior to an IHD death was higher in individuals with SCZ compared to others 23, but not significantly different between individuals with or without BD 29. Importantly, when restricting the analysis to people who were previously diagnosed with IHD, SCZ was only modestly associated with higher IHD mortality, which indicates that premature cardiovascular deaths in individuals with SMI may be prevented if CVDs were identified and treated 30, 31, 32. To clarify reasons for premature cardiovascular deaths in individuals with SMI, it is important to investigate to what extent these individuals are in contact with and treated in primary or specialized somatic health care prior to cardiovascular death. SCZ and BD often have an early onset and a chronic course, which makes it more likely to assume that the presence of SMI may have affected the detection and treatment of CVD in the years, possibly decades, leading up to a cardiovascular death. We chose not to merge SCZ and BD into one SMI group, as they often differ with regard to symptom expression, socioeconomic background of patients, and healthcare utilization. Previous studies 23, 29, 33 did not differentiate between sexes, although sex is associated with severity of both SMI 34, 35, 36 and CVD 37, and healthcare utilization 38, 39. We therefore present sex‐specific analyses, to facilitate identification of groups at particularly risk of fatal undiagnosed CVD.

### Aims of the study

We examined whether individuals with and without schizophrenia or bipolar disorder had equal likelihood of not being diagnosed with cardiovascular disease prior to cardiovascular death. Secondary aims were to describe sex‐specific differences between individuals with schizophrenia and bipolar disorder, and to describe demographic characteristics, comorbidity, and healthcare utilization in individuals with schizophrenia or bipolar disorder and undiagnosed cardiovascular disease prior to death.

## Material and methods

### Data sources

We obtained mortality data for the period 2011–2016 from the Norwegian Cause of Death Registry (CDR), and diagnostic data on individuals with SMI and/or CVD and information on use of primary and specialized health care from the Norwegian Directorate of Health's system for control and payment of health reimbursements in primary care (the KUHR database) and the Norwegian Patient Registry (NPR) for the period 2008–2016.

Nearly all healthcare services in Norway are publicly funded, which ensures representative and almost complete data in national health registries. The CDR provides 98% complete information on causes of death based on standardized certificates completed by the physician who examined the deceased 40. The CDR also receives notifications of autopsy findings and may request supplementary information from physicians, healthcare institutions, and clinical registries such as the Medical Birth Registry and the Cancer Registry. In approximately half of all cases, the underlying cause of death is determined using automated classification software 16, 17, which process data according to the rules of ICD‐10, whereas the remaining cases are set by professional coders (supervised by physicians), based on the death certificate and any supplementary information.

The KUHR database contains information on consultations in primary care (i.e., visits at general practitioners’ (GP) offices, home visits by GPs, and emergency room visits). As GPs are funded mainly on a fee‐for‐service basis, the completeness of reported contacts in the KUHR database is considered to be almost 100%. The NPR contains information on all contacts in specialized health care in Norway (i.e., government‐owned hospitals and out‐patient clinics, publicly financed substance use treatment facilities, and private health clinics with governmental reimbursement). Since 2008, the coverage in the NPR has been almost 100%, except for substance use treatment facilities which were included from 2009, and private somatic health clinics with governmental reimbursement, in which approximately 85% of all contacts were reported in the study period 41, 42.

Diagnostic codes in the CDR and the NPR follow the International Classification of Diseases and Related Health Problems, 10th Revision (ICD‐10), whereas diagnostic codes for primary care contacts in the KUHR database follow the International Classification of Primary Care 2nd Edition (ICPC‐2). Accurate linkage across data sources was obtained using an encrypted personal identification number included in all registries.

### Subject inclusion criteria and diagnostic categories

Deceased individuals aged 18 years or older were included if a diagnosis of CVD (ICD‐10 codes I00–I82) was recorded as underlying cause of death in the death certificate. The study included deaths in the years 2011–2016, securing at least 3 years of observation time in the NPR and the KUHR database prior to death (as information concerning healthcare utilization during 2008–2016 was included).

Cardiovascular causes of death were subdivided into IHD (I20–I25), other forms of heart disease (OHD, I30–I52) and cerebrovascular diseases (I60–I69). IHD was further subdivided into myocardial infarction (MI, ICD‐10 codes I21–I22 and I25.2), and OHD was further subdivided into heart failure (ICD‐10, codes I09.9, I11.0, I13.0, I13.2, I25.5, I42.0, I42.5–I42.9, I43, and I50) and arrhythmia (ICD‐10, codes I44.1–I44.3, I45.6, I45.9, and I47–I49). Foreign citizens and individuals registered as having died abroad were excluded (*N* = 132).

Individuals were included in the SMI group if a diagnosis of SCZ (ICD‐10 code F20 or ICPC‐2 code P72) or BD (ICD‐10 codes F30–F31 or ICPC‐2 code P73) was recorded in the NPR or the KUHR database during the years 2008–2016, or in the death certificate. Individuals diagnosed with both SCZ and BD (*N* = 97) were included in the SCZ group only.

A diagnosis of CVD was considered present if ICD‐10‐codes I00–I82 or G45, or corresponding ICPC‐2 diagnoses (codes K70–K71, K74–K80, K82–K84, K86–K87, and K89–K94) were recorded in the NPR or the KUHR database in the period from inclusion (January 1, 2008) and up to 1 month prior to death. Diagnoses of CVD recorded only within the last month prior to death were not counted to avoid including CVDs secondary to other fatal diseases.

### Analysis and statistical methods

Sex, age, and use of primary and specialized somatic care (the latter including all contacts in government‐owned hospitals, out‐patient clinics, and private health clinics with governmental reimbursement with a somatic diagnosis), and comorbidity up to 1 month prior to death were used to assess group comparability. Mean score on the Charlson comorbidity index (CCI) 43 was used to describe somatic comorbidity recorded in primary or specialized somatic care, and was modified to exclude CVD (i.e., MI, heart failure, peripheral vascular disease, and cerebrovascular disease), applying weights from Quan et al. 44. Continuous variables, with the exception of CCI, were presented as medians with the 25 and 75 percentile and compared using the Kruskal–Wallis test. Chi‐square tests were used to compare proportions. Significant results were followed by post hoc tests with Bonferroni correction for multiple comparisons to assess whether there were any differences between the three groups 45. A two‐tailed *P*‐value < 0.05 was considered statistically significant.

We applied the following analytic strategy: First, the proportion of people who had not been diagnosed with CVD prior to cardiovascular death was computed and compared across the three groups. Second, crude sex‐specific odds ratios (ORs) for not being diagnosed with CVD prior to cardiovascular death were calculated using logistic regression and reported with corresponding 95% confidence intervals (CI). Third, we adjusted for age at death adding a linear variable with six categories (age 18–49, 50–59, 60–69, 70–79, 80–89, and 90 years or above). No linear interactions between age and diagnostic groups were found. Sex‐ and age‐stratified ORs were also calculated, applying age‐groups 18–59, 60–79, and 80 years or above. Finally, we included in the model the modified mean score on the CCI and recorded substance use disorders (SUD, see diagnostic codes in Table S1). Subgroup analyses were conducted for individuals with IHD, MI, OHD, or cerebrovascular disease as underlying cause of death. To test the consistency of study findings, we also conducted additional analysis to assess the impact of (i) excluding individuals with dementia (see codes in Table S1), (ii) excluding cases with causes of death that should not be considered underlying causes of death 46, (iii) including CVD diagnosis during the last month prior to death, (iv) applying CVD recorded in specialized somatic care only as dependent variable, (v) including cases with CVD as contributing cause of death, and (vi) adjustment for observation time. All analyses were performed using sas statistical software, version 9.4 (SAS Institute Inc., Cary, NC, USA).

### Ethics

All patient data were fully de‐identified when accessed by the investigators. In Norway, studies with de‐identified information from medical health registries do not require participant consent. Legal basis and exemption from professional secrecy requirements for the use of personal health data in research were granted by the Regional Committee for Medical and Health Research Ethics (2014/72/REK nord).

## Results

### Characteristics of the study population

A total of 72 451 Norwegian citizens aged 18 years or older who died due to CVD in the period 2011–2016 were included in the study (Table 1). Of these, 814 (1.1%) were registered with SCZ and 673 (0.9%) with BD. During the study period, most individuals with SCZ (55%) had their SCZ diagnosis recorded in primary care only, while 35% had a SCZ diagnosis recorded in both primary and specialized health care, 8% in specialized health care only, and 3% in the CDR only (results not shown in the tables). Among individuals with BD, 37% had a BD diagnosis in primary care only, 47% both in primary and specialized health care, 15% in specialized health care only, and 1% in the CDR only.

**Table 1:** Cardiovascular deaths among individuals with schizophrenia, bipolar disorder, or no severe mental illness who died at ages 18 years or above, according to sex, age, and cause of death

	Schizophrenia	Bipolar disorder	No severe mental illness	*P*‐value	*Post hoc* comparisons
Men					
Deaths, *n*	384	291	33 015		
Age at death, mean (SD)	70.9 (14.3)	70.9 (13.0)	79.8 (12.1)	<0.0001	SCZ, BD < No SMI
Age 18–59 at death, *n* (%)	80 (20.8)	57 (19.6)	2330 (7.1)	<0.0001	No SMI < SCZ, BD
Age 60–79 at death, *n* (%)	181 (47.1)	154 (52.9)	10 853 (32.9)	<0.0001	No SMI < SCZ, BD
Age ≥ 80 at death, *n* (%)	123 (32.0)	80 (27.5)	19 832 (60.1)	<0.0001	SCZ, BD < No SMI
Underlying cause of death, *n* (%)					
I20–I25 Ischemic heart diseases	175 (45.6)	117 (40.2)	13 969 (42.3)	0.311	–
Myocardial infarction	102 (26.6)	65 (22.3)	8510 (25.8)	0.381	–
I30–I52 Other forms of heart disease	100 (26.0)	68 (23.4)	8422 (25.5)	0.676	–
Heart failure	45 (11.7)	28 (9.6)	3863 (11.7)	0.542	–
Arrhythmia	23 (6.0)	8 (2.7)	1988 (6.0)	0.064	–
I60–I69 Cerebrovascular diseases	70 (18.2)	71 (24.4)	6825 (20.7)	0.128	–
Other cardiovascular diseases^a^	39 (10.2)	35 (12.0)	3799 (11.5)	0.689	–
Women					
Deaths, *n*	430	382	37 949		
Age at death, mean (SD)	80.5 (13.3)	79.3 (12.6)	86.7 (9.5)	<0.0001	BD < SCZ < No SMI
Age 18–59 at death, n (%)	42 (9.8)	26 (6.8)	761 (2.0)	<0.0001	No SMI < BD < SCZ
Age 60–79 at death, n (%)	117 (27.2)	132 (34.6)	5446 (14.4)	<0.0001	No SMI < SCZ < BD
Age ≥ 80 at death, *n* (%)	271 (63.0)	224 (58.6)	31 742 (83.6)	<0.0001	SCZ, BD < NO SMI
Underlying cause of death, *n* (%)					
I20–I25 Ischemic heart diseases	152 (35.3)	135 (35.3)	11 883 (31.3)	0.064	–
Myocardial infarction	92 (21.4)	87 (22.8)	7532 (19.8)	0.310	–
I30–I52 Other forms of heart disease	123 (28.6)	101 (26.4)	12 004 (31.6)	0.040	–
Heart failure	60 (14.0)	58 (15.2)	5797 (15.3)	0.768	–
Arrhythmia	25 (5.8)	14 (3.7)	3162 (8.3)	0.001	BD < No SMI
I60–I69 Cerebrovascular diseases	114 (26.5)	96 (25.1)	9696 (25.6)	0.852	–
Other cardiovascular diseases^a^	41 (9.5)	50 (13.1)	4366 (11.5)	0.286	–

BD, bipolar disorder; SCZ, schizophrenia; SD, standard deviation; SMI, severe mental illness.

a Other cardiovascular diseases: ICD‐10 codes I00–I15, I26–I28, and I70–I82.

Women were in majority in all three groups (SCZ, BD, and no SMI; Table 1). Men and women with SMI died 9 and 7 years younger, respectively, than men and women without SMI. Nearly 75% of individuals without SMI died at ages 80 years or above, whereas less than half of individuals with SMI who died of CVD reached this age. IHD was the most common cause of cardiovascular death and accounted for nearly 40% of all deaths in the three groups. No significant differences in causes of cardiovascular deaths were noted across groups.

Men with SCZ had lower frequency of GP visits and specialized somatic out‐patient visits, but similar frequency of emergency room visits and somatic admissions, compared to men without SMI, whereas women with SCZ had higher frequency of emergency room visits, lower frequency of specialized somatic out‐patient visits, but similar frequency of GP visits and somatic hospital admissions compared to women without SMI (Table 2). Individuals with BD had higher frequency of both primary and specialized somatic care compared to individuals with SCZ, and also higher frequency of GP visits, emergency room visits, and somatic hospital admissions compared to individuals without SMI. Individuals with SMI had higher frequency of SUD compared to individuals without SMI, and women with SMI also higher prevalence of diagnosed chronic obstructive pulmonary disease (COPD) and dementia compared to women without SMI (Table 2).

**Table 2:** Healthcare utilization and comorbidity in individuals with schizophrenia, bipolar disorder, or no severe mental illness who died from CVD at ages 18 years or above, according to sex

	Schizophrenia	Bipolar disorder	No severe mental illness	*P*‐value	*Post hoc* comparisons
Men	384	291	33 015		
Patients according to healthcare sector, *n* (%)					
General practitioner	366 (95.3)	290 (99.7)	32 058 (97.1)	0.004	SCZ, No SMI < BD
Emergency room	283 (73.7)	240 (82.5)	23 570 (71.4)	0.000	SCZ, No SMI < BD
Specialized somatic care	337 (87.8)	278 (95.5)	30 880 (93.5)	<0.0001	SCZ < BD, No SMI
Specialized mental care	248 (64.6)	200 (68.7)	2840 (8.6)	<0.0001	No SMI < SCZ, BD
No healthcare use	5 (1.3)	0 (0.0)	478 (1.4)	0.115	–
No. of healthcare contacts per person‐year, median (25/75 percentile)					
GP visits	6.3 (3.0–12.3)	9.6 (5.6–15.2)	7.6 (3.9–13.3)	<0.0001	SCZ < No SMI < BD
Emergency room visits	0.5 (0.3–1.0)	0.7 (0.3–1.5)	0.5 (0.3–0.9)	<0.0001	SCZ, No SMI < BD
Somatic admissions	0.5 (0.2–1.0)	0.7 (0.3–1.2)	0.6 (0.3–1.1)	0.001	SCZ, No SMI < BD
Somatic out‐patient visits	1.0 (0.3–2.3)	2.1 (0.8–3.6)	2.0 (0.8–3.9)	<0.0001	SCZ < BD, No SMI
Patients with selected comorbidities, *n* (%)					
Diabetes	90 (23.4)	74 (25.4)	7551 (22.9)	0.568	–
COPD	65 (16.9)	65 (22.3)	5775 (17.5)	0.092	–
Substance use disorder	55 (14.3)	72 (24.7)	1846 (5.6)	<0.0001	No SMI < SCZ < BD
Dementia	77 (20.1)	66 (22.7)	5901 (17.9)	0.058	–
Modified CCI, mean (SD)	1.11 (1.8)	1.35 (1.8)	1.24 (1.7)	0.017	SCZ < BD, No SMI
No CC groups, *n* (%)	217 (56.5)	129 (44.3)	15 996 (48.5)	0.003	BD, No SMI < SCZ
≥2 CC groups, *n* (%)	71 (18.5)	69 (23.7)	7042 (21.3)	0.243	–
Women	430	382	37 949		
Patients according to healthcare sector, *n* (%)					
General practitioner	405 (94.2)	375 (98.2)	36 221 (95.4)	0.175	–
Emergency room	338 (88.0)	302 (79.1)	27 983 (84.8)	0.005	–
Specialized somatic care	399 (92.8)	368 (96.3)	35 898 (94.6)	0.082	–
Specialized mental care	251 (58.4)	240 (62.8)	3134 (8.3)	<0.0001	No SMI < SCZ, BD
No healthcare use	5 (1.2)	1 (0.3)	579 (1.5)	0.110	–
No. of healthcare contacts per person‐year, median (25/75 percentile)					
GP visits	6.4 (2.9–12.5)	9.3 (5.0–16.3)	7.1 (3.6–12.4)	<0.0001	SCZ, No SMI < BD
Emergency room visits	0.5 (0.3–1.1)	0.6 (0.3–1.1)	0.5 (0.3–0.9)	<0.0001	No SMI < SCZ, BD
Somatic admissions	0.5 (0.3–0.9)	0.7 (0.3–1.2)	0.5 (0.3–1.0)	0.000	SCZ, No SMI < BD
Somatic out‐patient visits	1.2 (0.5–2.3)	1.5 (0.7–3.1)	1.5 (0.6–2.9)	<0.0001	SCZ < No SMI < BD
Patients with selected comorbidities, *n* (%)					
Diabetes	92 (21.4)	82 (21.5)	6902 (18.2)	0.061	–
COPD	78 (18.1)	78 (20.4)	4805 (12.7)	<0.0001	No SMI < SCZ, BD
Substance use disorder	17 (4.0)	45 (11.8)	748 (2.0)	<0.0001	No SMI < SCZ < BD
Dementia	158 (36.7)	123 (32.2)	10 006 (26.4)	<0.0001	No SMI < SCZ, BD
Modified CCI, mean (SD)	1.25 (1.6)	1.37 (1.6)	1.08 (1.5)	<0.0001	No SMI < BD
No CC groups, *n* (%)	204 (47.4)	154 (40.3)	19 518 (51.4)	<0.0001	BD < No SMI
≥2 CC groups, *n* (%)	80 (18.6)	82 (21.5)	6227 (16.4)	0.016	No SMI < BD

BD, bipolar disorder; CC groups, Charlson Comorbidity groups; COPD, chronic obstructive pulmonary disease; GP, general practitioner; Modified CCI, Charlson comorbidity index modified to exclude CVD (e.g., myocardial infarction, heart failure, peripheral vascular disease, and cerebrovascular disease); SCZ, schizophrenia; SD, standard deviation; SMI, severe mental illness.

### Undiagnosed CVD prior to cardiovascular death

**Figure 1:**
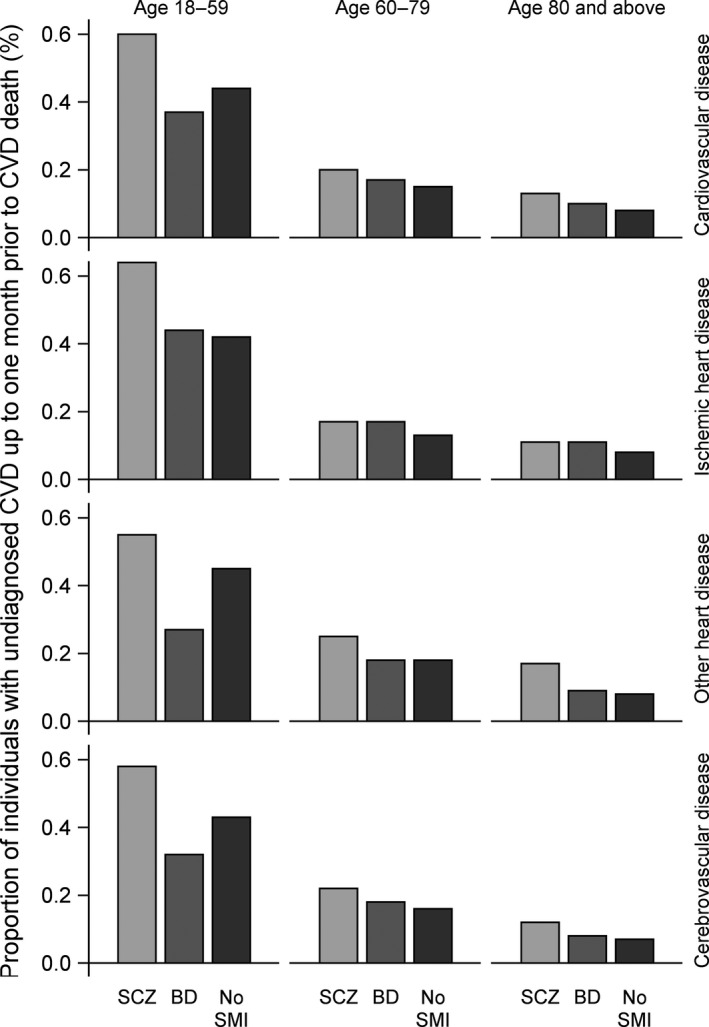
Proportion of individuals with schizophrenia, bipolar disorder, and no severe mental illness (no SMI) who were undiagnosed with cardiovascular disease up to one month prior to cardiovascular death, according to cause of death, age at death, and patient group.

Twenty‐three per cent of individuals with SCZ who died from CVD had not been diagnosed with CVD in primary or specialized somatic health care prior to death, compared to 17% in individuals with BD and 11% in individuals without SMI (*P* < 0.0001). The highest proportion of undiagnosed CVD prior to cardiovascular death was found in individuals who died at ages 18–59, of whom 60% of individuals with SCZ, 37% of individuals with BD, and 44% of individuals without SMI were undiagnosed with CVD prior to death (Fig. 1).

Most individuals with diagnosed CVD prior to death had their CVD diagnosis recorded both in primary and specialized somatic care, but individuals with SCZ least so (74% in individuals with SCZ, compared to 79% in individuals with BD and 80% in individuals without SMI, *P* = 0.012, results not shown in the tables). A slightly higher proportion of individuals with SCZ had their CVD diagnosis recorded only in specialized somatic health care prior to death (11% in individuals with SCZ, compared to 9% of individuals with BD, and 6% of individuals without SMI, *P* = 0.050, results not shown in tables).

**Figure 2:**
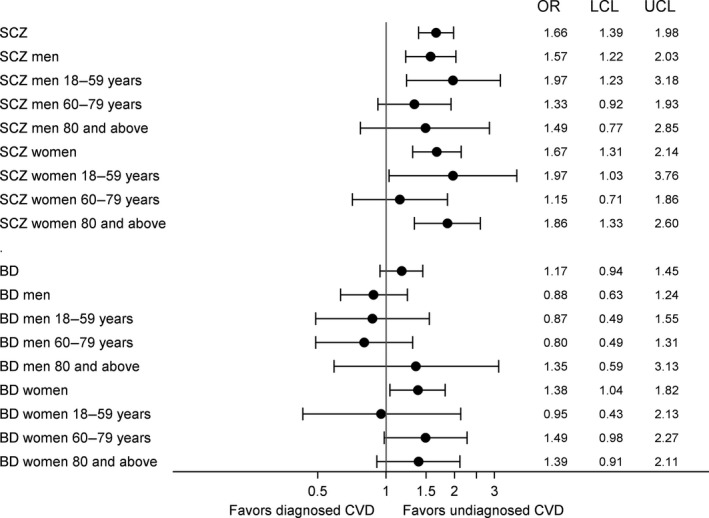
Adjusted odds ratios with 95% upper (UCL) and lower (UCL) confidence limits for not being diagnosed with cardiovascular disease prior to cardiovascular death in individuals with schizophrenia or bipolar disorder, according to sex and age‐group.

Unadjusted analyses showed that individuals with SCZ or BD had 132% and 56% higher odds, respectively, of not being diagnosed with CVD prior to cardiovascular death, compared to individuals without SMI (OR 2.32; 95% CI: 1.97–2.74 in individuals with SCZ and OR: 1.56; 95% CI: 1.27–1.92 in individuals with BD, results not shown in tables). After adjustment for age at death and comorbidities, we found that individuals with SCZ were 66% more likely (OR: 1.66; 95% CI: 1.39–1.98), women with BD were 38% more likely (OR: 1.38; 95% CI: 1.04–1.82), and men with BD were equally likely (OR: 0.88, 95% CI: 0.63–1.24) not to be diagnosed with CVD prior to cardiovascular death, compared to individuals without SMI (Fig. 2). Recorded SUD and higher medical comorbidity were negatively associated with the odds of undiagnosed CVD (results not shown), but inclusion of these covariates did not alter results notably compared to the age‐adjusted models (Table 3).

**Table 3:** Subgroup analyses showing odds ratios with 95% confidence intervals for not being diagnosed with CVD prior to cardiovascular death, according to sex, patient group, cause of death, and model specifications

	Crude	Adjusted for age^a^	Adjusted for age, SUD, and comorbidity^b^
	OR (95% CI)^c^	OR (95% CI)^c^	OR (95% CI)^c^
Both genders			
Ischemic heart disease			
Schizophrenia	**2.40** (1.87–3.07)	**1.64** (1.26–2.12)	**1.70** (1.30–2.22)
Bipolar disorder	1.23 (0.88–1.73)	0.84 (0.59–1.19)	0.92 (0.64–1.31)
Myocardial infarction			
Schizophrenia	**2.49** (1.83–3.39)	**1.85** (1.33–2.56)	**1.99** (1.42–2.78)
Bipolar disorder	1.37 (0.92–2.05)	1.05 (0.69–1.60)	1.14 (0.74–1.74)
Other forms of heart disease (I30–I52)			
Schizophrenia	**2.69** (1.91–3.79)	**1.82** (1.27–2.61)	**1.85** (1.28–2.67)
Bipolar disorder	**1.67** (1.06–2.65)	1.00 (0.62–1.61)	1.08 (0.67–1.76)
Cerebrovascular			
Schizophrenia	**1.77** (1.23–2.54)	1.43 (0.99–2.06)	**1.47** (1.01–2.13)
Bipolar disorder	**1.74** (1.19–2.54)	1.38 (0.93–2.03)	1.45 (0.98–2.15)
Men			
Ischemic heart disease			
Schizophrenia	**2.40** (1.72–3.34)	1.42 (0.99–2.03)	**1.47** (1.02–2.11)
Bipolar disorder	1.24 (0.76–2.00)	0.70 (0.42–1.16)	0.78 (0.47–1.31)
Myocardial infarction			
Schizophrenia	**2.00** (1.30–3.09)	1.40 (0.87–2.23)	1.48 (0.92–2.38)
Bipolar disorder	**1.65** (0.93–2.90)	1.09 (0.60–2.00)	1.20 (0.65–2.21)
Other forms of heart disease (I30–I52)			
Schizophrenia	**3.20** (1.95–5.26)	1.73 (0.99–3.02)	1.73 (0.98–3.06)
Bipolar disorder	1.95 (0.96–3.96)	0.90 (0.43–1.90)	0.98 (0.46–2.09)
Cerebrovascular			
Schizophrenia	**2.78** (1.62–4.78)	**1.80** (1.02–3.16)	1.75 (0.99–3.09)
Bipolar disorder	1.32 (0.67–2.58)	0.91 (0.45–1.81)	0.96 (0.47–1.92)
Women			
Ischemic heart disease			
Schizophrenia	**2.41** (1.67–3.50)	**1.85** (1.26–2.72)	**1.91** (1.29–2.83)
Bipolar disorder	1.26 (0.78–2.03)	0.94 (0.58–1.53)	0.99 (0.61–1.62)
Myocardial infarction			
Schizophrenia	**3.19** (2.06–4.93)	**2.47** (1.57–3.89)	**2.68** (1.68–4.27)
Bipolar disorder	1.20 (0.68–2.14)	0.97 (0.54–1.74)	1.03 (0.57–1.86)
Other forms of heart disease (I30–I52)			
Schizophrenia	**2.34** (1.46–3.76)	**1.82** (1.12–2.96)	**1.85** (1.13–3.03)
Bipolar disorder	1.50 (0.82–2.76)	1.06 (0.57–1.96)	1.14 (0.61–2.14)
Cerebrovascular			
Schizophrenia	1.30 (0.80–2.11)	1.13 (0.69–1.85)	1.23 (0.75–2.03)
Bipolar disorder	**2.04** (1.28–3.24)	**1.68** (1.05–2.71)	**1.77** (1.09–2.87)

CI, confidence interval; OR, odds ratio; SUD, substance use disorder.

a Adjusted for age‐group at death (categories 18–49, 50–59, 60–69, 70–79, 80–89, and 90 and above).

b Adjusted for age‐group at death (see note †), SUD, and comorbidities (modified Charlson Index).

c Bold figures: Significant association at *P*‐value < 0.05.

Subgroup analyses according to cause of death with adjustment for age at death and comorbidities showed that individuals with SCZ had higher odds of not being diagnosed with CVD prior to death for all main causes of cardiovascular death (Table 3). Women with SCZ had particularly high odds of undiagnosed CVD prior to a MI death. Individuals with BD had similar odds of undiagnosed CVD prior to cardiovascular death as individuals without SMI, except that women with BD who died from cerebrovascular disease had higher odds of undiagnosed CVD (Table 3).

The sensitivity analyses (Figure S1) confirmed the main results; SCZ (in both men and women) and BD in women were associated with increased odds of not being diagnosed with CVD prior to CVD death, whereas no association was found for BD in men.

### Characteristics of individuals with undiagnosed CVD

Individuals with SMI who died from undiagnosed CVD died approximately 10 years younger than individuals with undiagnosed CVD without SMI (Table S2). Individuals with SMI and undiagnosed CVD had lower likelihood of dying in a nursing home, and individuals with SCZ and undiagnosed CVD had a higher likelihood of death at home, compared to individuals without SMI. No differences with regard to cause of death were found between the three groups (results not shown).

Almost all (98%) individuals with SMI and undiagnosed CVD had visited primary or specialized somatic health care prior to death, compared to 88% among individuals without SMI (results not shown in tables). Eighty‐eight per cent of individuals with SCZ and 97% of individuals with BD had visits in primary care in the observation period, compared to 78% of individuals without SMI, and a majority of individuals with SMI had also been in contact with specialized somatic health care prior to death (73% of individuals with SCZ, and 84% in individuals with BD, compared to 70% of individuals without SMI). Having no healthcare contacts up to 1 month prior to death was uncommon in individuals with SMI (5% and 1% in individuals with SCZ or BD, respectively), while this was the case for 12% of individuals without SMI. Individuals with BD and undiagnosed CVD prior to death had more GP contacts and more admissions in specialized somatic care, compared to individuals without SMI who died from undiagnosed CVD, and individuals with SMI more emergency room visits (Table S2).

Compared to individuals with SMI and diagnosed CVD prior to death, individuals with SMI and undiagnosed CVD prior to death died younger, more often at home (SCZ only), less often in a nursing home, more often at places outside home and healthcare institutions (BD only), and more often from IHD (SCZ only) (Table S2). They also had fewer healthcare contacts in primary care, fewer admissions and out‐patient visits in specialized somatic care prior to death, and more recorded SUD than individuals with SMI and diagnosed CVD.

## Discussion

In this study based on the entire Norwegian population, we found that men and women with SCZ and women with BD were more likely to be undiagnosed with CVD prior to cardiovascular death, even though most had been in contact with primary or specialized somatic care prior to death. The higher odds of undiagnosed CVD applied to all main causes of cardiovascular death and was most pronounced in the youngest.

The higher level of undiagnosed CVD prior to cardiovascular death in individuals with SCZ is in accordance with earlier studies reporting doubled risk of unforeseen death in individuals with SCZ, CVD being the most common cause 33, and also in accordance with studies reporting a decreased likelihood in individuals with SCZ of being diagnosed with somatic illness in the early courses of diseases 24, 47. It is also in accordance with a Swedish registry study, in which the proportion of individuals who died from IHD undiagnosed up to 1 month prior to an IHD death was higher in individuals with SCZ, compared to individuals without SCZ 23. Our study extends these earlier findings by documenting that the higher likelihood of undiagnosed CVD prior to death in individuals with SCZ may be related to the low age at death, and by documenting that the higher likelihood of undiagnosed CVD prior to death in individuals with SCZ applies to all main causes of cardiovascular death and is particularly pronounced for deaths from MI in women with SCZ. Our finding of similar odds of undiagnosed CVD prior to an IHD death in individuals with BD is also in accordance with earlier findings 29, but the increased odds of undiagnosed CVD prior to a cerebrovascular death in women with BD is a novel finding. Earlier studies have found an increased incidence of vascular disease in women with BD, but not in men with BD 48, 49. An increased risk of adverse illness course in women with BD has been reported 34 and may also contribute to this finding. We also observed higher levels of somatic out‐patient visits in men with BD, including a higher level of somatic out‐patient visits with a CVD diagnosis (*P* = 0.001, results not shown), indicating increased healthcare seeking and/or increased referral rates to specialized somatic care in men with BD, compared to women with BD. The finding may not be a stable finding, however, and should be replicated in independent studies.

We do not assume that cardiovascular deaths always can be prevented, as they may result from sudden and unpredictable events such as MI and stroke. Nevertheless, differences between groups found for the same causes of death in our study suggest that other mechanisms are also at work, both at individual, provider, and system level. Current risk prediction algorithms for CVD have been shown to underestimate the risk in individuals with SCZ, particularly in younger men 50, and may likely lead to lower detection rates in individuals with SCZ compared to others. Also, unclear responsibility with regard to physical health examination in patients with SMI 51, complexity of care and misinterpretation of physical complaints as psychosomatic symptoms 52, as well as separation between primary and secondary health care and between somatic and psychiatric health care, may contribute to underdiagnosis. General stigma toward people with SMI 53, particularly those with comorbid SUD, has also been reported 54, although our results showed a negative association between recorded SUD and undiagnosed CVD. Given the overlap in genetic risk factors for SMI and CVD 16, it could be that individuals with SMI are more susceptible to CVD. Genetic risk for a severe outcome would under other circumstances imply an increased alertness in clinicians. Under these assumptions, reduced access to screening and treatment would have a disproportionate effect in the SMI population.

The ability to recognize somatic symptoms and seek timely somatic care may be impaired in individuals with SMI due to negative symptoms, cognitive impairment, and social isolation. Increased pain tolerance 55, as well as increased risk of silent CVD 56 not associated with traditional cardiovascular risk factors 57, has also been reported in individuals with SCZ, possibly affecting healthcare seeking. Reduced or delayed healthcare seeking has been reported earlier for older non‐affective psychotic patients, especially regarding CVD 58.

In our study, undiagnosed CVD was associated with young age at death. Individuals with SCZ have increased the prevalence of risk factors for early cardiovascular mortality 59 and are at increased risk of dying at ages when healthcare providers may not usually suspect CVD. There is also a possibility that younger patients are prescribed higher doses of antipsychotic medicine due to more severe symptoms at an early stage in the disease course and hence are at higher risk of adverse cardiac effects such as arrhythmias, deviations in blood pressure, heart failure, myocarditis, and sudden death 60, 61, 62, 63, 64, 65. Both typical and atypical antipsychotic medication have been associated with sudden cardiac death 66, 67, through mechanisms such as QTc interval lengthening 68, possibly leading to fatal arrhythmias, and myocarditis and cardiomyopathy associated with clozapine use 69. The possibility of more lethal, or faster progressing, CVD in individuals with SMI may also influence the odds for not having CVD diagnosed prior to cardiovascular death.

### Strengths and limitations

The main strength of this study is the use of unselected, complete nationwide data on all individuals who died of CVD during a 6‐year period, including complete diagnostic data from both primary and specialized health care, and individuals without any healthcare contacts in the observation period prior to death. However, some limitations need attention. First, we had no information concerning history of CVD prior to the study period. Stable, long‐term and currently untreated CVDs may thus have led to misclassification of undiagnosed CVD in this study. As individuals with SMI have higher lethality from CVD 70, 71, 72, our findings probably underestimate the real difference between individuals with and without SMI. It could be, however, that people with SMI to a larger extent live with, for example, untreated valve insufficiency, which is not treated due to contraindications to major surgery (such as smoking), although newer, less invasive techniques may have reduced this potential disparity. We did not have information on prescription of cardiovascular medication, but it is unlikely that such prescriptions would not be accompanied by a CVD diagnosis in primary care.

Second, we lacked information on antipsychotic prescriptions, which made us unable to study the association between antipsychotic medication and sudden cardiac death. We observed, however, a similar (SCZ) or lower (BD) likelihood of fatal arrhythmias in individuals with SMI, compared to individuals without SMI.

Third, we had no information on the severity of CVD. However, as the study included only deceased individuals, control for differences in medical need may be approximated by the fact that they all died from the same underlying causes of death. Also, studies of undiagnosed MI in the general population 73 and in individuals with SMI 71 have reported similar demographic characteristics, similar coronary risk profiles, and similar mortality among persons with diagnosed and previously undiagnosed MI.

Fourth, the validity of diagnoses is a concern when using administrative registry data. Previous studies have found high agreement between DSM‐IV diagnosis and clinical diagnoses of SMI in the NPR 74, and valid information regarding stroke 75. Also, in other Nordic registries, the validity of cardiovascular diagnoses in general 76, 77, atrial fibrillation and atrial flutter 78, and intracerebral hemorrhage 79 have been found to be high, whereas the validity of heart failure diagnoses 80 and peripheral arterial disease diagnoses 81 may be questionable. The accuracy of diagnoses in the KUHR database has not yet been established. According to Statistics Norway, about 0.3% of main diagnoses set by the GP in the KUHR database in 2015 were assumed to have an incorrect diagnostic code 82, and the validity of diagnostic coding in general practice registries in other countries has been found good, particularly for chronic diseases 83. We included persons with a diagnosis of affective disorder (ICPC‐2 code P73) in the BD group, which may have led to an inclusion of individuals with severe depression but not BD.

The CDR is found to provide high‐quality information on causes of death 84. Autopsy is considered as the gold standard for determination of causes of death, but is infrequently undertaken in Norway. During the period 2008–2013, the proportion of autopsies in Norway remained stable at 7.5%, equally distributed between medical and forensic autopsies 85. A Norwegian study from 2011 reported very good agreement between autopsy findings and mortality statistics for both stroke and coronary heart disease in the CDR 86. An earlier Norwegian study investigating to what extent underlying cause of death based on the death certificate was changed when taking into account autopsy results, found a substantial (17%) underreporting of cardiovascular deaths, particularly in the young and old, and in women 87. The decision to perform an autopsy is, however, not made at random, and findings from such studies may therefore not be directly transferable to other cases. The most common error is that immediate or intermediate causes of death are registered as the underlying cause of death 88. We found, however, similar results when we included all deaths with CVD as underlying or contributing cause of death, as shown in the sensitivity analysis. We had no data on sudden death (ICD‐10 code R98), possibly implying underestimated cardiovascular deaths. However, only about 2% of underlying causes of death in Norway in the years 2011–2016 were assigned to unknown or unspecified causes (R96–R99) 7. Longer postmortem time before discovery in individuals with SCZ compared to others has also been reported 89, possibly affecting the reliability of cause of death codes in individuals with SCZ.

Fifth, although it is possible to enter at least three diagnoses per primary care contact, 85% of these encounters had only one recorded diagnosis, both in individuals with and without SMI. There is therefore a possibility that only the SMI diagnosis was recorded, even if CVD may have been the subject of the consultation. However, among individuals with SMI, only 17% of the primary healthcare encounters included a SMI diagnosis, so it seems reasonable to assume that any recognized CVD would be recorded at some point in the observation period. Also, analysis with recorded CVD in specialized health care only as dependent variable gave similar results, as shown in the sensitivity analysis.

Furthermore, individuals with SMI have a substantially increased risk of premature death from suicides and accidents, particularly in the youngest age‐groups 10, 90. As persons with SMI included in our study must have lived long enough to develop CVD, this may have introduced survivor bias. Substance abuse and low socioeconomic status, which are associated with increased risk of accidental death, and also increased CVD risk, were, however, not associated with increased risk of undiagnosed IHD 23, 29 or increased risk of unforeseen death 33 in earlier studies.

Finally, the findings are representative of individuals with severe CVD in countries with publicly funded and readily available health services for both somatic and mental disorders.

## Clinical implications

Persons with SMI constitute a subgroup at particularly high risk of early and severe CVD 50. In our study, almost all individuals with SMI with undiagnosed CVD prior to cardiovascular death utilized primary or specialized somatic care in the observation period preceding death, showing that opportunities for identification and management of cardiovascular disease exist. Hence, a diagnosis of SCZ or BD should act as a trigger for enhanced screening for CVD among the youngest patients, and antipsychotic medicine in younger patient groups should be carefully selected in order to minimize cardiovascular risk. Furthermore, there is a need to define more accurately the responsibility for maintaining follow‐up and treatment of CVD risk factors and early CVD in patients with SMI. Albeit death from undiagnosed CVD cannot always be prevented, the results from our study indicate that improved health care at a structural level should be specifically directed toward younger patients. Improved recognition, monitoring, and treatment of individual CVD risk factors should be stressed. Further research is strongly needed, to investigate age‐differentiated risk factors and interventions both at the system level and at the individual level.

## Declaration of interest

None.

## Supporting information


**Figure S1.** Results of sensitivity analyses.
**Table S1.** List of diagnoses describing psychiatric comorbidity.
**Table S2.** Characteristics of deaths, health care utilization and comorbidity in the subgroups with undiagnosed and diagnosed CVD prior to cardiovascular death.
