# Supplementary material for: Undiagnosed cardiovascular disease prior to cardiovascular death in individuals with severe mental illness
Source: Acta Psychiatr Scand. 2019 Mar 29;139(6):558–71. doi: 10.1111/acps.13017 (PMC6619029; doi:10.1111/acps.13017)
Supplement: Supplementary file 1 — Figure S1. Results of sensitivity analyses. Table S1. List of diagnoses describing psychiatric comorbidity. Table S2. Characteristics of deaths, health care utilization and comorbidity in the subgroups with undiagnosed and diagnosed CVD prior to cardiovascular death. [file ACPS-139-558-s001.docx]

**Supporting Information**

Table S1. List of diagnoses describing psychiatric comorbidity

| Variable | ICD-10 code | ICPC-2 code | Description |
| --- | --- | --- | --- |
| Dementia | F00 |  | Dementia in Alzheimer disease |
|  | F01 |  | Vascular dementia |
|  | F02 |  | Dementia in other diseases classified elsewhere |
|  | F03 |  | Unspecified dementia |
|  | F05.1 |  | Delirium superimposed on dementia |
|  | G30 |  | Alzheimer disease |
|  | G31.1 |  | Senile degeneration of brain, not elsewhere classified |
|  |  | P70 | Dementia |
| Substance use related disorders | F10-16, F18-F19 | | Mental and behavioral disorders due to use of psychoactive substances, except tobacco (F17) |
|  | E24.4 |  | Alcohol-induced pseudo-Cushing syndrome |
|  | E52 |  | Niacin deficiency [pellagra] |
|  | G31.2 |  | Degeneration of nervous system due to alcohol |
|  | G62.1 |  | Alcoholic polyneuropathy |
|  | G72.1 |  | Alcoholic myopathy |
|  | I42.6 |  | Alcoholic cardiomyopathy |
|  | K29.2 |  | Alcoholic gastritis |
|  | K70 |  | Alcoholic liver disease |
|  | K86.0 |  | Alcohol-induced chronic pancreatitis |
|  | O35.4 |  | Maternal care for (suspected) damage to fetus from alcohol |
|  | O35.5 |  | Maternal care for (suspected) damage to fetus by drugs |
|  | Z50.2 |  | Alcohol rehabilitation |
|  | Z50.3 |  | Drug rehabilitation |
|  | Z71.4 |  | Alcohol abuse counselling and surveillance |
|  | Z71.5 |  | Drug abuse counselling and surveillance |
|  | Z72.1 |  | Alcohol use |
|  | Z72.2 |  | Drug use |
|  |  | P15 | Chronic alcohol abuse |
|  |  | P16 | Acute alcohol abuse |
|  |  | P18 | Medication abuse |
|  |  | P19 | Drug abuse |

Table S2. Characteristics of deaths, health care utilization and comorbidity in the subgroups with undiagnosed and diagnosed CVD prior to cardiovascular death.

|  |  |  |  | Schizophrenia | | Bipolar disorder | | No severe mental illness | | p-value | Post hoc comparisons |
| --- | --- | --- | --- | --- | --- | --- | --- | --- | --- | --- | --- |
| Individuals with undiagnosed CVD prior to CVD death | | | | | | | | | | | |
|  | *Deaths, n* | | | 186 |  | 112 |  | 8,020 |  |  |  |
|  | *Age at death, mean (SD)* | | | 66.1 | (17.0) | 68.5 | (14.7) | 76.8 | (16.4) | <.0001 | SCZ, BD < No SMI |
|  |  | Age 18-59 at death, n (%) | | 73 | (39.2) | 31 | (27.7) | 1,351 | (16.8) | <.0001 | No SMI < BD < SCZ |
|  |  | Age 60-79 at death, n (%) | | 61 | (32.8) | 50 | (44.6) | 2,475 | (30.9) | 0.007 | No SMI < BD |
|  |  | Age ≥ 80 at death, n (%) | | 52 | (28.0) | 31 | (27.7) | 4,194 | (52.3) | <.0001 | SCZ, BD < No SMI |
|  | *Place of death, n (%)* | | |  |  |  |  |  |  |  |  |
|  |  | Home | | 63 | (33.9) | 32 | (28.6) | 1,833 | (22.9) | 0.001 | No SMI < SCZ |
|  |  | Hospital | | 46 | (24.7) | 41 | (36.6) | 2,378 | (29.7) | 0.093 | - |
|  |  | Nursing home | | 54 | (29.0) | 26 | (23.2) | 3,051 | (38.0) | 0.000 | SCZ, BD < no SMI |
|  |  | Other or unknown | | 23 | (12.4) | 13 | (11.6) | 758 | (9.5) | 0.309 | - |
|  | *Patients according to health care sector, n (%)* | | | | |  |  |  |  |  |  |
|  |  | GP or emergency room | | 164 | (88.2) | 109 | (97.3) | 6,313 | (78.7) | <.0001 | No SMI < SCZ, BD |
|  |  | Specialized somatic care | | 135 | (72.6) | 94 | (83.9) | 5,589 | (69.7) | 0.004 | No SMI < BD |
|  |  | No health care use | | 9 | (4.8) | 1 | (0.9) | 986 | (12.3) | <.0001 | SCZ, BD < No SMI |
|  | *Health care utilization per person-year, median (IQR)* | | | | |  |  |  |  |  |  |
|  |  | GP visits | | 3.7 | (0.9-7.5) | 7.3 | (3.1-13.5) | 1.7 | (0.2-4.7) | <.0001 | No SMI < SCZ < BD |
|  |  | Emergency room visits | | 0.2 | (0.0-0.5) | 0.2 | (0.0-0.6) | 0.0 | (0.0-0.3) | <.0001 | No SMI < SCZ, BD |
|  |  | Somatic admissions | | 0.0 | (0.0-0.23) | 0.2 | (0.0-0.5) | 0.0 | (0.0-0.20) | <.0001 | No SMI < SCZ < BD |
|  |  | Somatic outpatient visits | | 0.3 | (0.0-1.2) | 0.8 | (0.2-2.1) | 0.4 | (0.0-1.3) | 0.001 | SCZ, No SMI < BD |
| Individuals with diagnosed CVD prior to CVD death | | | | | | | | | | | |
|  | *Deaths, n* | | | 628 |  | 561 |  | 62,944 |  |  |  |
|  | *Age at death, mean (SD)* | | | 78.9 | (12.4) | 77.1 | (12.7) | 84.3 | (10.2) | <.0001 | BD < SCZ < No SMI |
|  |  | Age 18-59 at death, n (%) | | 49 | (7.8) | 52 | (9.3) | 1,740 | (2.8) | <.0001 | No SMI < SCZ, BD |
|  |  | Age 60-79 at death, n (%) | | 237 | (37.7) | 236 | (42.1) | 13,824 | (22.0) | <.0001 | No SMI < SCZ, BD |
|  |  | Age ≥ 80 at death, n (%) | | 342 | (54.5) | 273 | (48.7) | 47,380 | (75.3) | <.0001 | SCZ, BD < No SMI |
|  | *Place of death, n (%)* | | |  |  |  |  |  |  |  |  |
|  |  | Home | | 110 | (17.5) | 136 | (24.2) | 9,821 | (15.6) | <.0001 | SCZ, No SMI < BD |
|  |  | Hospital | | 173 | (27.5) | 176 | (31.4) | 21,825 | (34.7) | 0.000 | SCZ < No SMI |
|  |  | Nursing home | | 319 | (50.8) | 228 | (40.6) | 29,006 | (46.1) | 0.002 | BD < SCZ, No SMI |
|  |  | Other or unknown | | 26 | (4.1) | 21 | (3.7) | 2,292 | (3.6) | 0.797 | - |
|  | *Patients according to health care sector, n (%)* | | | | |  |  |  |  |  |  |
|  |  | GP or emergency room | | 607 | (96.7) | 556 | (99.1) | 61,966 | (98.4) | 0.001 | SCZ < BD, No SMI |
|  |  | Specialized somatic care | | 601 | (95.7) | 552 | (98.4) | 61,189 | (97.2) | 0.017 | SCZ < BD |
|  |  | No health care use | | 1 | (0.2) | 0 | (0.0) | 71 | (0.1) | 0.685 | - |
|  | *Health care utilization per person-year, median (IQR)* | | | | |  |  |  |  |  |  |
|  |  | GP visits | | 6.9 | (3.1-13.6) | 9.7 | (5.5-16.0) | 7.7 | (4.1-13.2) | <.0001 | SCZ < No SMI < BD |
|  |  | Emergency room visits | | 0.4 | (0.1-1.0) | 0.5 | (0.2-1.1) | 0.3 | (0.1-0.7) | <.0001 | NO SMI < SCZ < BD |
|  |  | Somatic admissions | | 0.5 | (0.3-1.0) | 0.6 | (0.3-1.1) | 0.5 | (0.2-1.0) | <.0001 | SCZ, No SMI < BD |
|  |  | Somatic outpatient visits | | 1.2 | (0.5-2.5) | 1.8 | (0.8-3.6) | 1.7 | (0.7-3.4) | <.0001 | SCZ < BD, No SMI |

Abbreviations: CVD, Cardiovascular disease; SCZ, Schizophrenia; BD, Bipolar disorder; SMI, Severe mental illness; GP, General practitioner; SD, Standard Deviation; IQR, Interquartile range.


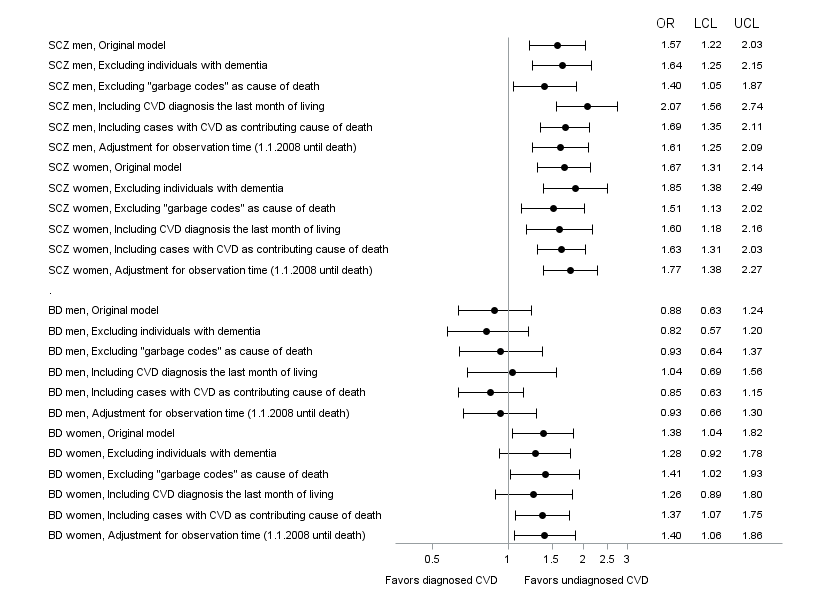
 Figure S1. Results of sensitivity analyses. Adjusted Odds Ratios (OR) with 95% upper (UCL) and lower (UCL) Confidence Limits for not being diagnosed with cardiovascular disease (CVD) prior to cardiovascular death in individuals with schizophrenia (SCZ) or bipolar disorder (BD), according sex, patient group and differing inclusion criteria.
